# Hyperbaric Oxygen Therapy during Pregnancy for Critical Anemia Secondary to Sickle Cell Disease with Post-Transfusion Hyperhemolysis: A Case Report

**DOI:** 10.3390/hematolrep16030053

**Published:** 2024-08-30

**Authors:** Shawn Khan, Connor T. A. Brenna, Jacob Pendergrast, A. Kinga Malinowski, Marcus Salvatori, Rita Katznelson, Jordan Tarshis

**Affiliations:** 1 Temerty Faculty of Medicine, University of Toronto, Toronto, ON M5S 1A8, Canada; shaw.khan@mail.utoronto.ca (S.K.); connor.brenna@mail.utoronto.ca (C.T.A.B.); jacob.pendergrast@uhn.ca (J.P.); ann.malinowski@sinaihealth.ca (A.K.M.); marcus.salvatori@uhn.ca (M.S.); rita.katznelson@uhn.ca (R.K.); 2 Department of Anesthesiology & Pain Medicine, University of Toronto, Toronto, ON M5G 0A8, Canada; 3 Department of Medical Oncology and Hematology, University Health Network, Toronto, ON M5G 2C4, Canada; 4 Department of Obstetrics and Gynaecology, University of Toronto, Toronto, ON M5G 1E2, Canada; 5 Department of Anesthesia, University Health Network, Toronto, ON M5G 2C4, Canada; 6 Hyperbaric Medicine Unit, Toronto General Hospital, Toronto, ON M5G 2C4, Canada; 7 Department of Anesthesia, Sunnybrook Health Sciences Centre, Toronto, ON M4N 3M5, Canada

**Keywords:** hyperbaric oxygen therapy, HBOT, pregnancy, sickle cell disease, hyperhemolysis syndrome

## Abstract

**Background:** Sickle cell disease is the most common human monogenetic disease, and its risks are amplified during pregnancy. **Methods**: This report describes a 35-year-old woman with HgbSS sickle cell disease who developed hyperhemolysis syndrome after undergoing an exchange transfusion during pregnancy. **Results:** In addition to conventional medical treatment, the patient received prepartum hyperbaric oxygen therapy (HBOT), totaling 17 treatments for the indication of severe anemia. She experienced significant clinical improvement while undergoing HBOT and ultimately delivered a healthy preterm infant by cesarean section. **Conclusions:** The risks, benefits, and challenges of using HBOT in this unique context are discussed.

## 1. Introduction

Sickle cell disease (SCD) is the most common human monogenetic disease and is characterized by red blood cells with an atypical propensity to assume an abnormal, rigid, sickle-like shape [1]. Sickling occurs as a result of a mutation in the hemoglobin (Hb) β chain, which leads to the polymerization of hemoglobin (HbS) when in a deoxygenated state. While administration of supplemental oxygen can decrease erythrocyte sickling, its clinical benefit as a treatment for sickle cell disease has not been well established [2].

Pregnancy is a uniquely high-risk state for patients living with SCD. Several adaptive physiological changes of pregnancy shift the hematologic balance in favor of vaso-occlusive events, such as states of hypercoagulability, venous stasis, and increased metabolic demands. Consequently, SCD is associated with higher rates of maternal mortality, miscarriage, preterm delivery, intrauterine growth restriction, stillbirth, and preeclampsia [3].

The following case report describes the management of a pregnant patient with HbSS SCD who experienced a vaso-occlusive crisis and a delayed transfusion reaction following the commencement of a prophylactic red blood cell exchange transfusion. Hyperbaric oxygen therapy (HBOT) was applied beginning during the second trimester, with the aim of reducing oxygen debt as well as temporizing her state of critical anemia to allow time for medical therapy to take effect.

## 2. Case Presentation

A 35-year-old gravida 3 para 2 woman presented to the emergency department at 15-week gestation with worsening pain in her legs and back. Her medical history was significant for HbSS SCD, with multiple previous vaso-occlusive crises. Existing sequelae of SCD included an L3 vertebral body infarct, longstanding splenomegaly with chronic thrombocytopenia, and chronic pain requiring long-term opioid therapy.

Two previous pregnancies were complicated by recurrent vaso-occlusive events requiring hospital admission as well as severe anemia causing fetal distress. In each prior pregnancy, she received two red cell transfusions in the context of non-resolving SCD crises and non-reassuring fetal heart rates. Both pregnancies resulted in the birth of healthy infants via cesarian section at 36- and 37-week gestational age, respectively.

Her blood type was O+ and her pre-pregnancy baseline hemoglobin on hydroxyurea ranged from 90 to 100 g/L. There was no history of alloimmunization or adverse transfusion reaction. Her most recent transthoracic echocardiogram, three years prior, showed a left ventricular ejection fraction (LVEF) of 64% and right ventricular systolic pressure (RVSP) of 19 mmHg.

Prior to this pregnancy, she had been stable on hydroxyurea with a HbF of 25% (Table 1). Per clinical guidelines, given concerns for teratogenicity, hydroxyurea was discontinued prior to conception [4]. Owing to the complex obstetrical history, the option of re-starting hydroxyurea in the second trimester of pregnancy was offered but declined by the patient. Instead, she was initiated on monthly two-unit manual exchange transfusions, a transfusion protocol expected to achieve similar levels of HbS% as had been achieved with hydroxyurea while minimizing the number of donor exposures.

**Figure 1:**
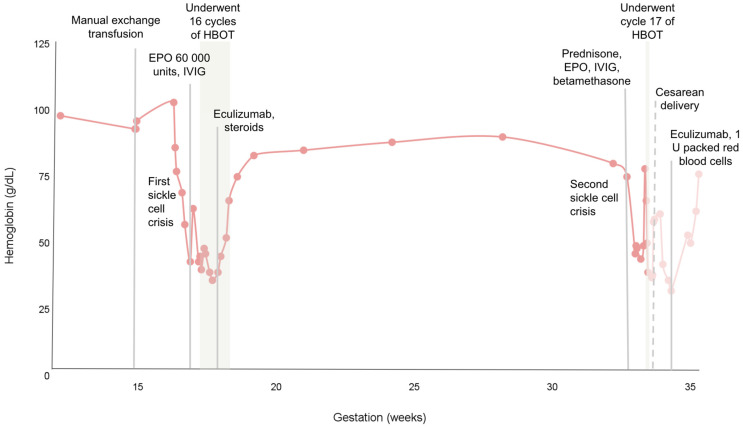
Trend of hemoglobin (g/dL) during pregnancy in relation to adjuvant therapies.

The first manual exchange transfusion was completed at 15 weeks of gestational age (see Figure 1). Her Hb prior to transfusion was 93 g/L, with a HbS percentage of 73.4%. Following the exchange transfusion, her Hb was 96 g/L, with a HbS of 56.7%. Given that she did not have a history of RBC alloimmunization, her prophylactic antigen matching was limited to Rh (D,C,c,E,e) and Kell antigens.

The patient presented to the hospital ten days post-transfusion with worsening pain in her legs and back and was admitted for a presumed vaso-occlusive event. Her Hb on admission was 103 g/L, but over the course of four days in the hospital, it dropped to 43 g/L, accompanied by signs of accelerating hemolysis and a lack of compensatory reticulocytosis (Table 2). Although there was no evidence of alloimmunization or serologic incompatibility with the red blood cells she had received 10 days prior, her hemoglobin electrophoresis demonstrated a rapid decrease in her HbA%, consistent with a delayed hemolytic transfusion reaction. As this was accompanied by a fall in her total hemoglobin to levels below what had been observed pre-transfusion, the hematology service made the diagnosis of hyperhemolysis syndrome (HHS). Notably, her HbF% fell only slightly despite cessation of hydroxyurea, reflecting a possible underlying diagnosis of hereditary persistence of fetal hemoglobin.

She was started urgently on high-dose intravenous immune globulin (IVIG) and erythropoietin (EPO). Due to concerns regarding the safety of pregnancy, high-dose steroids and eculizumab were initially deferred, and she was instead referred for HBOT in an attempt to optimize oxygen delivery to her and her fetus.

The initial HBOT was delivered with 100% oxygen in a monoplace chamber at 2.8 atmospheres absolute (ATA), reduced to 2.0 ATA for the final 30 min to minimize the risk of oxygen toxicity, for a total duration of 90 min with 2 air breaks. During these treatments, the patient experienced both subjective and objective clinical improvement, with decreased breathing and respiratory rates. In light of this, she continued to receive HBOT at 2.8 ATA for 90 min twice daily, with at least 8 h between treatments. Serial laboratory values and vital signs are presented in Table 2 and Table 3.

The patient experienced no adverse effects attributable to HBOT but did require oral and IV opioids before and after treatments to manage ongoing thigh pain. Her target Hb was >50 g/L and she received a total of 16 sessions. During this time, she was also administered high-dose methylprednisolone and eculizumab. Her hemolytic markers and reticulocyte count gradually improved, and she was eventually discharged from the hospital clinically stable and without fetal distress, with a Hb of 83 g/L.

At 32 + 6 weeks gestation, the patient presented again with symptomatic anemia and severe pain centered around her right ribs and both shoulders, minimally responsive to hydromorphone and acetaminophen, and was readmitted with a Hb that dropped from 75 g/L to 44 g/L over 48 h. She developed jaundice and choluria and began experiencing irregular contractions albeit with a stable fetal heart rate (FHR) tracing. She was re-started on prednisone, erythropoietin, and IVIG, and also received betamethasone at this time for fetal lung maturation in anticipation of a preterm delivery (see Figure 1).

HBOT was again considered for critical anemia, balancing the potential benefits with the risks of daily absences from the labor ward without FHR monitoring for several hours per session; however, there were few other available treatment options given her prior hyperhemolytic reaction, and with informed consent, HBOT was reinitiated once daily with a plan for FHR monitoring for the 30 min preceding and following each session. The predetermined endpoints of treatment include the following: (1) any non-reassuring FHR tracings, (2) hemoglobin remaining less than 50 g/L after repeated treatments, or (3) a need for an urgent cesarian section.

She received only one treatment session (at 33 + 3 weeks gestation) before developing pre-eclampsia, and the following day she underwent an emergent cesarian section. At that time, her Hb was 66 g/L. The infant was born at 33 + 4 weeks gestational age with a weight of 2.2 kg (50th percentile for gestational age) and APGAR scores of 3, 2, and 7 at 1, 5, and 10 min, respectively. The placenta had no significant pathology, and its weight was between the 25th and 50th percentile.

Despite minimal post-partum blood loss, her hemoglobin fell again to 37 g/L, with progressive signs of hemolysis and a falling reticulocyte count. Another two doses of eculizumab were administered. Following the subsequent development of worsening airspace disease, hypoxemia, and fever, a unit of RBCs was transfused for the treatment of acute chest syndrome. Due to the high degree of immunosuppression she had already undergone, this transfusion was not preceded by rituximab, but the patient’s steroid dose was increased from prednisone 75 mg daily to methylprednisolone 1 g. While this resulted in a sustained increase in her hemoglobin, her postoperative course was further complicated, despite thromboprophylaxis, by a subsegmental right lower lobe pulmonary embolus and non-occlusive thrombi in the internal iliac veins bilaterally. Therapeutic anticoagulation was initiated, and, despite the subsequent development of abdominal wall and pelvic hematomas, her hemoglobin remained stable. HBOT was not resumed postpartum despite a drop in Hb as she did not demonstrate evidence of acute organ ischemia.

In follow-up at three months postpartum, this patient’s Hb was 75 g/L, with an improving reticulocyte count. She has been restarted on hydroxyurea with improvement in pain symptoms, and her infant is developing normally.

## 3. Discussion

Exchange transfusion is a proposed strategy for managing complex SCD in pregnancy, as hydroxyurea is often stopped due to animal study-based teratogenicity concerns. However, as observed in this case, transfusions are not without risk, and when hyperhemolysis develops, it may be refractory to immunosuppressive therapies. HBOT was used in this case as an adjunct to medical treatment to relieve whole-body oxygen debt and provide time for these other medical treatments to take effect.

HBOT involves the delivery of 100% oxygen at supra-atmospheric pressures in specialized hyperbaric chambers [5]. The increased pressure causes a large increase in dissolved plasma oxygen, resulting in increased oxygen tissue delivery independent of Hb. HBOT itself has some risks, including barotrauma to the ears and sinuses, acceleration of pre-existing cataracts, reversible myopia, and oxygen toxicity [6], and requires careful safety protocols to eliminate the risk of fire in an enclosed high-oxygen environment.

HHS is poorly understood but well-recognized in SCD. The proposed pathophysiology includes bystander complement-mediated hemolysis, macrophage-activation syndrome, and ineffective erythropoiesis [7]. As is the case in up to one-third of HHS cases, there was no evidence of underlying serologic incompatibility with the RBC units transfused during this patient’s second trimester of pregnancy. HBOT is known to have immunosuppressive and anti-inflammatory effects, including inhibition of IL-1 from macrophages [8].

Eculizumab is a recombinant humanized monoclonal antibody that binds to the human C5 complement protein and inhibits the activation of terminal complement. It has been suggested that, as SCD is a chronic hemolytic condition, the complement activation that occurs during a crisis may exacerbate hemolysis (bystander hemolysis), and this activation can be decreased with eculizumab [9].

HBOT for SCD in pregnancy is uncommon, likely due to limited access to hyperbaric oxygen facilities in close proximity to labor wards. However, there are case reports offering separate descriptions of HBOT for SCD and HBOT during pregnancy. In 1981, Molzhaninov et al. managed 170 pregnant women with heart disease using HBOT. This approach successfully counteracted hypoxia in both mothers and their fetuses, and no complications were reported [10]. Vanina et al. described HBOT during pregnancy in a series of 54 patients with cardiopulmonary pathology resulting in arterial hypoxia, as indicated by pO_2_ levels below 70 mmHg. Two women and three infants died due to the underlying disease, but again with no reports of complications due to HBOT [11]. In 2001, Abboud et al. documented two cases of third-trimester maternal carbon monoxide poisoning. Both patients underwent HBOT and were subsequently delivered at term. One of the newborns was diagnosed with an antenatal ischemic cerebral lesion, possibly attributable to carbon monoxide poisoning [12].

HBOT has not previously been implicated as a trigger for pre-eclampsia. In this case, the patient was diagnosed with pre-eclampsia within 24 h of a single session of HBOT when it was restarted at 33 + 3 weeks of gestation. This event raises the question as to whether HBOT exposure in late gestation, in the context of critical ischemia and a high output state, could potentially induce cardiovascular changes favorable for the development of maternal type 1 pre-eclampsia. However, a single case report cannot provide evidence of this type of effect.

HBOT has also been used successfully to treat SCD vaso-occlusive crises [13] and the relatively common complication of avascular necrosis [14]. The mechanism of benefit appears to be the relief of whole-body hypoxia and not a direct effect on RBC morphology, as Mychaskiw et al. reported no effects on RBC morphology in an in vitro study of SCD blood aliquots [15].

The effects of hyperbaric oxygen on erythropoiesis are not well described, but there is a suggestion in the literature that extrahepatic erythropoiesis is inhibited by hyperoxic conditions [16]. The patient described in this case received exogenous EPO, which may have compensated for any decrease in endogenous erythropoietin.

In the complex case reported here, it is challenging to definitively attribute changes in the patient’s clinical course to HBOT in the context of concomitant medical therapies and the natural history of HHS over time. The rarity of this clinical scenario similarly challenges comparison to other modes of treatment: few reports describe the management of peripartum HHS in the context of SCD, detailing complicated prenatal courses despite standard medical therapy and cesarian deliveries at 28 weeks [17] and 34 weeks of gestation [18,19]. As a single case study, the present report is limited with respect to its generalizability to other practice settings and cannot establish causality. However, the clinical improvements in this patient during treatments and the biologically plausible mechanism support further consideration of HBOT as a temporizing measure in scenarios such as this. Balancing the risks and benefits for this patient population is challenging and requires a multidisciplinary team of providers experienced in high-risk obstetrical care, transfusion medicine, and hyperbaric medicine, as well as a hyperbaric medicine facility in proximity to the high-risk antenatal ward and the ability to rapidly intervene for an emergent obstetrical delivery should the need arise.

## Figures and Tables

**Table 1:** Baseline hemoglobin concentrations.

	Hb (g/L)	Plts (10^9^/L)	HbA (%)	HbA2 (%)	HbS (%)	HbF (%)
1 year prior	105	78	0	2.3	72	25.7
Pre-pregnancy	98	81	0	2.4	73.4	24.2

**Table 2:** Trend of complete blood count and hemolysis markers during the first hospital stay.

	Hb (g/L)	Plts (10^9^/L)	HbA (%)	HbA2 (%)	HbS (%)	HbF (%)	Retic (10^9^/L)	LDH	Bilirubin (µmol/L)
Pre-admission day 9	93	NR	NR	2.4	73.4	24.2	NR	NR	NR
Pre-admission day 9	96	90.6	22.5	2.4	56.7	18.4	255	NR	24
Admission	103	84	NR	NR	NR	NR	NR	NR	31
PAD1	77	68	NR	NR	NR	NR	224	475	NR
PAD2	69	65	15	3.2	63	19.3	222	580	63
PAD3	57	63	NR	NR	NR	NR	NR	NR	65
PAD4	43	74	17.8	3.0	58	20.7	209	739	100
PAD5	63	127	NR	NR	NR	NR	NR	673	86
PAD6	43	80	12.6	3.5	NR	20.0	189	639	76
PAD7	40	95	13	2.9	NR	19.8	272	NR	65
PAD8	46	135	NR	NR	NR	NR	307	694	NR

HbA, hemoglobin A; HbS, hemoglobin S; Hb, hemoglobin; LDH, lactate dehydrogenase; NR, not reported; PAD, post-admission day; Plts, platelet count; Retic, reticulocytes.

**Table 3:** Patient vital signs before and after hyperbaric oxygen therapy.

	Blood Pressure (mmHg)		Heart Rate (bpm)		SpO_2_ (%)	
	Pre-HBOT	Post-HBOT	Pre-HBOT	Post-HBOT	Pre-HBOT	Post-HBOT
PAD6	122/73	123/80	121	103	NR	NR
PAD7 AM	120/68	123/79	123	98	94	98
PAD8 PM	137/76	123/83	115	97	96	97
PAD9 AM	122/68	123/75	104	98	93	98
PAD9 PM	126/65	134/86	117	96	98	100

HBOT, hyperbaric oxygen therapy; SpO_2_, oxygen saturation; PAD, post-admission day.

## Data Availability

All the data is contained within the case report.
